# 2187 The role of community in an evolving community-university pilot award program

**DOI:** 10.1017/cts.2018.265

**Published:** 2018-11-21

**Authors:** Sarah Wiehe, Gina E. M. Claxton, Lisa Staten, Ann Alley, Eric Beers, Elaine Lipscomb

**Affiliations:** 1 Indiana University School of Medicine; 2 Office of Primary Care, Indiana State Department of Health; 3 Indiana Healthy Weight Initiative, Indiana Public Health Association

## Abstract

OBJECTIVES/SPECIFIC AIMS: To fulfill the Indiana Clinical and Translational Sciences Institute’s (Indiana CTSI) Community Health Partnerships’ (CHeP) mission of improving the health of Indiana residents through community-university partnerships, CHeP engaged with community partners to develop and implement a pilot award program for community-based participatory research, the Trailblazer Award (TA). The objective is to describe the engagement processes throughout the pilot program timeline and as the pilot program evolved over the 6-year period since the program started. METHODS/STUDY POPULATION: Though a process of engagement with community stakeholders, we assessed the process for each year of the TA, noting what changes occurred and how they occurred. Engagement for the TA process occurred during the following phases: RFA development, review, active project support, dissemination of project results, and project/partnership follow-up. RESULTS/ANTICIPATED RESULTS: During the RFA development phase, we decided to focus the award on health equity for 5 years; and we implemented structural changes to encourage new partnerships in underrepresented and rural areas. During the review phase, we incorporated both community and university reviewers and co-moderators. To increase capacity among our reviewer pool, we offered webinars and repeated opportunities to serve as reviewers. During the project support phase, we added the following: community-based CITI training; opportunities for networking with peer awardee teams; and community and academic co-led sessions on addressing recruitment barriers, grant writing, and dissemination to a community audiences. Through our active engagement of the CHeP Advisory Board, one Board member (from Indiana State Department of Health) leveraged matching funds for the TA, effectively doubling the number of projects supported each year. DISCUSSION/SIGNIFICANCE OF IMPACT: Whereas previous work has reported on engagement during the review process of pilot award applications, we discuss ways to extend engagement to include other aspects of a pilot program both before and after the review process. In our process, several key partners offered insightful changes that have resulted in a more engaged program.

